# *Angiostrongylus costaricensis* infection in Martinique, Lesser Antilles, from 2000 to 2017

**DOI:** 10.1051/parasite/2018022

**Published:** 2018-04-10

**Authors:** Céline Dard, Duc Nguyen, Charline Miossec, Katia de Meuron, Dorothée Harrois, Loïc Epelboin, André Cabié, Nicole Desbois-Nogard

**Affiliations:** 1 Laboratoire de Parasitologie-Mycologie-Sérologies Bactériennes et Parasitaires, CHU de la Martinique, 97200 Fort-de-France France; 2 Laboratoire de Parasitologie-Mycologie, CHU Grenoble Alpes, 38700 Grenoble France; 3 Institute for Advanced Biosciences (IAB), INSERM U1209 − CNRS UMR5309, Université Grenoble Alpes, 38700 Grenoble France; 4 Service de Maladies Infectieuses et Tropicales et Médecine Polyvalente, CHU de la Martinique, 97200 Fort-de-France France; 5 EA3593, Ecosystèmes Amazoniens et Pathologie Tropicale, Université de la Guyane, 97306 Cayenne France; 6 Service de Pédiatrie, Maison de la Femme de la Mère et de l'Enfant, 97200 Fort-de-France France; 7 Laboratoire de Biologie Médicale, Centre Hospitalier de Basse-Terre, 97109 Basse-Terre, Guadeloupe France; 8 Unité des Maladies Infectieuses et Tropicales, Centre Hospitalier Andrée Rosemon, 97306 Cayenne France; 9 CIC Antilles-Guyane, INSERM 1424, Centre Hospitalier Andrée Rosemon, 97306 Cayenne France; 10 Université des Antilles, EA4537, 97200 Fort-de-France France

**Keywords:** *Angiostrongylus costaricensis*, Abdominal angiostrongyliasis, helminth, intestinal parasitosis, eosinophilic ileocolitis, Martinique, French Antilles, Lesser Antilles, Caribbean

## Abstract

Human abdominal angiostrongyliasis (HAA) is a parasitic disease caused by the accidental ingestion of the nematode *Angiostrongylus costaricensis* in its larval form. Human infection can lead to severe ischemic and inflammatory intestinal lesions, sometimes complicated by life-threatening ileal perforations. Only one case had been reported in Martinique, an Island in the French Antilles, in 1988. We retrospectively reviewed the medical charts of patients diagnosed with abdominal angiostrongyliasis at the University Hospital of Martinique between 2000 and 2017. The objectives of this study were to evaluate the incidence and perform a descriptive analysis of the clinical, biological, radiological, and histopathological features of HAA in Martinique. Two confirmed cases and two probable cases were identified in patients aged from 1 to 21 years during the 18-year period, with an estimated incidence of 0.2 cases per year (0.003 case/year/100.000 inhabitants (IC95% = 0.00–0.05)). All patients presented with abdominal pain associated with high blood eosinophilia (median: 7.24 G/L [min 4.25; max 52.28 G/L]). Two developed ileal perforation and were managed by surgery, with diagnostic confirmation based on histopathological findings on surgical specimens. The other two cases were probable, with serum specimens reactive to *Angiostrongylus* sp. antigen in the absence of surgery. All cases improved without sequelae. The description of this case series highlights the need to increase awareness of this life-threatening disease in the medical community and to facilitate access to specific diagnostic tools in Martinique. Environmental and epidemiological studies are needed to broaden our knowledge of the burden of this disease.

## Abstract (fr)

L'angiostrongylose abdominale humaine (AAH) est une maladie parasitaire causée par l'ingestion accidentelle du nématode *Angiostrongylus costaricensis* sous sa forme larvaire. L'infection humaine peut conduire à des lésions intestinales ischémiques et inflammatoires sévères, parfois compliquées par des perforations iléales menaçant le pronostic vital. Un seul cas avait été signalé en Martinique, une île des Antilles françaises, en 1988. Nous avons revu rétrospectivement les dossiers médicaux des patients ayant reçu un diagnostic d'angiostrongylose abdominale au CHU de la Martinique entre 2000 et 2017. Les objectifs de cette étude étaient d'évaluer l'incidence et effectuer une analyse descriptive des caractéristiques cliniques, biologiques, radiologiques et histopathologiques de l'AAH en Martinique. Deux cas confirmés et deux cas probables ont été identifiés chez des patients âgés de 1 à 21 ans au cours de la période de 18 ans, avec une incidence estimée à 0,2 cas par an (0,003 cas / an / 100 000 habitants (IC95% = 0,00 − 0,05)). Tous les patients présentaient une douleur abdominale associée à une éosinophilie sanguine élevée (médiane: 7,24 G/L [min 4,25; max 52,28 G / L]). Deux ont développé une perforation iléale et ont été traités par chirurgie, avec une confirmation diagnostique basée sur les résultats histopathologiques sur des échantillons chirurgicaux. Les deux autres cas étaient probables, avec des échantillons sériques réagissant aux antigènes d'*Angiostrongylus* sp. en l'absence de chirurgie. Tous les cas se sont améliorés sans séquelles. La description de cette série de cas souligne la nécessité de sensibiliser davantage la communauté médicale à cette maladie potentiellement mortelle et de faciliter l'accès à des outils diagnostiques spécifiques en Martinique. Des études environnementales et épidémiologiques sont nécessaires pour élargir nos connaissances sur cette parasitose.

## Introduction

Human abdominal angiostrongyliasis (HAA) is a zoonotic disease caused by a nematode, *Angiostrongylus costaricensis* Morera & Céspedes, 1971 [44]. The definitive hosts are rodents of the Cricetidae, Heteromyidae, and Muridae families [19,40,60,62]. Adult nematodes reside in the mesenteric arterial system of wild rodents, in which females lay eggs that generate first-stage larvae (L1), which are shed in the rodents' feces. Larval maturation to the third-stage (L3) occurs in intermediate hosts, mainly slugs from the families Veronicellidae and Limacidae [11,21,46,62]. Human infection is accidental and occurs by ingesting third-stage larvae (L3) from mollusks or vegetables contaminated with their slime [43]. Once ingested, the larvae invade intestinal tissues, reach sexual maturity, and release eggs in the ileo-cecal mesenteric arteries, causing eosinophilic enteritis in humans [66].

*A. costaricensis* was first discovered in the mesenteric arteries of humans in Costa Rica in 1967 [7,45], followed by the description of adult worms in the rodent *Sigmodon hispidus* in 1971 [44]. *A. costaricensis* is now found from Texas [64] southward to Argentina [52], including Honduras [27,58], Venezuela [23,69], Mexico [70], Brazil [71], Colombia [35], Nicaragua [12], El Salvador [68], Ecuador [30], Guatemala [28], Panama [63], Peru, [60] and probably French Guiana [65]. The disease is a public health problem in South America, in particular in Costa Rica, where it affects 12/100,000 persons, with approximately 500 new cases each year [43]. Some sero-epidemiological studies in South America have shown strong seroprevalence rates in humans, *i.e*., from 29.8 to 66.0% in endemic areas of Southern Brazil. This implies numerous asymptomatic infections [17] and a far broader distribution of the parasite in the Americas than previously believed [34]. In contrast, HAA is rarely reported in the Antilles and only six sporadic cases have been described since 1974 (Table 2). Among them, one was reported in Martinique, an island in the French Antilles.

The main objective of this study was to evaluate the incidence of symptomatic HAA cases in Martinique. The secondary objective was to perform a descriptive analysis of the clinical, biological, radiological, and histopathological features of these cases.

## Materials and Methods

### Setting

Martinique is a French Overseas Department in the Antilles, with a population of 380,877 inhabitants as of January 1^st^, 2015 (INSEE census, www.insee.fr). It has a tropical climate, with a rainy season from June to November and a dry season from December to May.

### Study design

A retrospective monocentric observational study was performed in the University Hospital of Martinique between January 1, 2000 and December 31, 2017. Data were extracted from the hospital data information system (PMSI), in which classification is based on the International Classification of Diseases, Tenth Revision (ICD-10). Hospital data codes for HAA (B813) were selected from the PMSI databases. Demographic data, abdominal imaging, biological results, clinical features, and outcomes were anonymously and retrospectively collected from the medical charts according to the legal and ethical guidelines of the French National Committee on Data Protection (CNIL). Serological assays to detect IgG against *Angiostrongylus* sp. were performed at the Swiss Tropical and Public Health Institute, Basel, Switzerland. Sera were first tested using the ELISA helminth screening test (detecting *Toxocara* sp., *Trichinella* sp., *Echinococcus* sp., *Fasciola* sp., Filaria, *Schistosoma* sp., and *Strongyloides* sp.) followed by a western blot using antigens derived from *A. cantonensis* adult worms [13].

### Case definition

We defined a confirmed case as a patient with clinical symptoms and biological results consistent with HAA (fever, abdominal tenderness, and blood eosinophilia) and histopathological findings of HAA (identification of worms, eggs, or larvae in the intestinal wall). A probable case was defined as a patient with clinical symptoms consistent with HAA and a serum specimen with IgG reactive to *Angiostrongylus* sp. antigen.

### Ethics statement

The variables were secondarily anonymized and retrospectively collected from medical charts. The French National Committee on Data Protection (CNIL)authorizes the retrospective use of anonymous patient files on the site of patient care in a single hospital.

## Results

During the 18-year period of the study, two confirmed and two probable cases of HAA were identified (male:female 50:50, median age: 7.5 years [min 1; max 21 years]). The annual incidence rate was 0.003 cases/100,000 habitants/year (95 CI% = 0.00–0.05). Most cases (75%) were diagnosed during the rainy season. All cases presented abdominal pain associated with high blood eosinophilia (median: 7.24 G/L [min 4.25; max 52.28 G/L]). The eosinophilia rate was not related to the severity of the disease. Cases 1 and 2, diagnosed in 12-month old children, were particularly severe and required surgical procedures with diagnostic confirmation by histological findings. These cases were characterized by anemia, a marked loss of weight and the presence of Charcot Leyden crystals in feces. Cases 3 and 4, probable, were diagnosed in a teenager and an adult with serum specimens reactive to *Angiostrongylus* sp. antigen. The length of hospitalization was variable (median 17.5 days [min 7; max 53 days]) and correlated with disease severity. All cases improved without sequelae. The clinical presentation along with the biological, imaging, histopathological, and epidemiological features are described in Table 1.   

**Table 1:** Clinical characteristics of the four patients with confirmed (cases 1 and 2) and probable (cases 3 and 4) *Angiostrongylus costaricensis* infection in Martinique. *A. fulica*: *Achatina fulica*, CRP: C-reactive protein, CSF: Cerebrospinal fluid, CT scan: Computerized axial tomography, Dx: x Days, EBV: Epstein-Barr virus, IV: intra-venous, *L. aurora*: *Limicolaria aurora*, ND: No Data.

Case	1	2	3	4
**Background data**				
**Year of diagnosis**	2000	2001	2016	2017
**Season**	August, rainy season	October, rainy season	November, rainy season	February, dry season
**Sex**	M	F	F	M
**Age**	12 months	12 months	14 years	21 years
**Area of residence (city, district)**	Le Lamentin	Saint-Esprit	Le Robert	Fort-de-France
**Living conditions**	Residential area	Residential area	Residential area	ND
**Medical history**	None	None	None	None
**Reported contact with mollusks**	None, but numerous slugs of und. species in the garden	None, but numerous slugs of und. species and snails (*A. fulica*, *L. aurora*,) in the garden	None, but numerous snails (*A. fulica*, *L. aurora*) at school	ND
**Clinical picture**				
**Duration of symptoms before admission**	1 month	2 weeks	1 month	24 hours
**Extra-digestive symptoms**	Irritability, moderate fever (38.5 °C) > 7 days	Decreased reactivity, fever (38.0 °C)>14 days	Fever>14 days;	Fever (39.0 °C)
**Loss of weight**	3.2% in 7 days (basal weight 9120 g)	6% in 15 days	None	None
**Digestive symptoms**	Anorexia, emesis, right iliac fossa pain, diarrhea, trails of blood in feces, dehydration	Anorexia, right iliac fossa pain, watery diarrhea, emesis	Severe right iliac fossa pain, emesis	Abdominal pain in suprapubic region, diarrhea, emesis
**Laboratory results**				
**Initial WBC** (10^9^/L)	25.5	19.1	19.8	63.3
**Max eosinophilia** (G/L (%))	4.25 (17)	4.68 (19)	9.8 (49.2)	52.28 (82.6)
**Initial hemoglobin** (g/dL)	9.6	6.8	15.5	15.5
**CRP** (mg/L)	95	94.5	3	223
**Liver, renal, hemostatic parameters**	Normal	Normal	Normal	Normal
**Microbiological analyses**	Blood, urine, CSF cultures: negative	Negative blood culture, positive urine culture (*E. coli* 10^5^ UFC/ml)	None	Blood and urine cultures: negative
***Angiostrongyluscantonensis* serology**	None	None	Positive (IgG, Western-Blot)	Positive (IgG, Western-Blot)
**Helminth ELISA screening test**^a^	None	None	Negative	Negative
**Other parasitic serology**	None	Schistosomiasis, toxocariasis: negative	Toxocariasis: negative	Schistosomiasis, toxocariasis: negative
**Parasitological examination of feces**	Few altered embryonated eggs of helminths & numerous Charcot Leyden crystals (D4 after surgery).	Negative, numerous Charcot Leyden crystals	Negative (3 times)	Negative (2 times)
**Abdominal imaging & surgery**				
**Abdominal imagery**	Ultrasound: dilated ileum, peritoneal exudate in the right iliac fossa, X-rays: distended left colic flexure (Fig. 1)	X-rays (Fig. 3): pneumoperitoneum under right hypochondria	Ultrasound: colon wall thickening, mild intraperitoneal effusion	CT scan: micronodular pulmonary pattern, peripheral lymphadenopathy.
**Exploratory laparotomy/ laparoscopy**	Laparotomy (D3): ischemic and congestive ileum, necrotic areas, mesenteric lymph node enlargement	Laparotomy (D50)	None	None
**Surgical procedure**	18 cm long ileal resection and anastomosis	16 cm long ileal resection (distal ileon + ileo-cecal valve) with 3 cm of healthy surgical resection margins and anastomosis	None	None
				
**Histology of resected specimen**				
**Macroscopic aspect**	Rigid, ulcerated, and hemorrhagic pattern	Surgical specimen agglutinated, necrotized, and covered with false membranes	None	None
**Histological examination of surgical specimen**	Polymorphic granulomas & eosinophilic infiltration of the intestinal mucosa, 60 to 80 μm long and mostly embryonated ovoid eggs within the granulomas with macrophages and eosinophils, thrombotic phenomena in muscular arteria caused by degenerated 140 to180-μm long *A. costaricensis* adults (Fig. 2A and 2B).	Ischemic intestinal wall, granulomas with giant cells, plasmocytes and eosinophilic cells, *A. costaricensis* eggs (Fig. 4A), larvae (Fig. 4B) and adults in the lumen of some vessels (Fig. 4C and 4D)	None	None
**Diagnosis & Medical care**				
**Diagnosis of angiostrongyliasis**	Histology of resected ileal specimen (D3 after hospitalization)	Histology of resected ileal specimen (D50 after hospitalization)	Probable with positive *A. cantonensis* serology (D30 after hospitalization)	Probable with positive *A. cantonensis* serology
**Concomitant infections**	None	EBV primary infection, urinary tract infection	None	None
**Symptomatic treatment and treatment for co-infections**	After surgery: blood transfusion, proper hydration, analgesia and nutrition, antibiotics (ceftriaxone, metronidazole)	IV antibiotics for urinary tract infection (cefotaxime, netilmicin), After surgery: blood transfusion, parenteral rehydration, antipyretics, antibiotics (cefotaxime, amikacin, metronidazole)	Acetaminophen, domperidone	Acetaminophen
**Anthelmintic treatment**	Thiabendazole 75 mg/kg/day (10 days)	Flubendazole empirical treatment (3 days) before diagnosis, thiabendazole 50 mg/kg/day (5 days) after diagnosis	Thiabendazole (5 days)	Ivermectin (18 mg in single dose)
**Length of hospitalization**	25 days	2 hospitalizations 1^st^: 16 days 2^nd^: 37 days	7 days	10 days
**Outcome**				
**Clinical improvement**	3 weeks after surgery	3 weeks after surgery	2 weeks after anthelmintic treatment	Regression of symptoms
**Decline of eosinophilia**	1.41 G/L D18 after hospitalization	1.17 G/L D71 after first hospitalization	0.40 G/L 10 months after hospitalization	2.0 G/L D80 after hospitalization
**Sequelae & clinical outcome**	Recovery	Recovery	Recovery	Recovery

a Helminth ELISA screening test simultaneously detects seven different species of tissue helminths (*Toxocara* sp., *Trichinella* sp., *Echinococcus* sp., *Fasciola* sp., Filaria, *Schistosoma* sp. and *Strongyloides* sp.).

**Figure 1:**
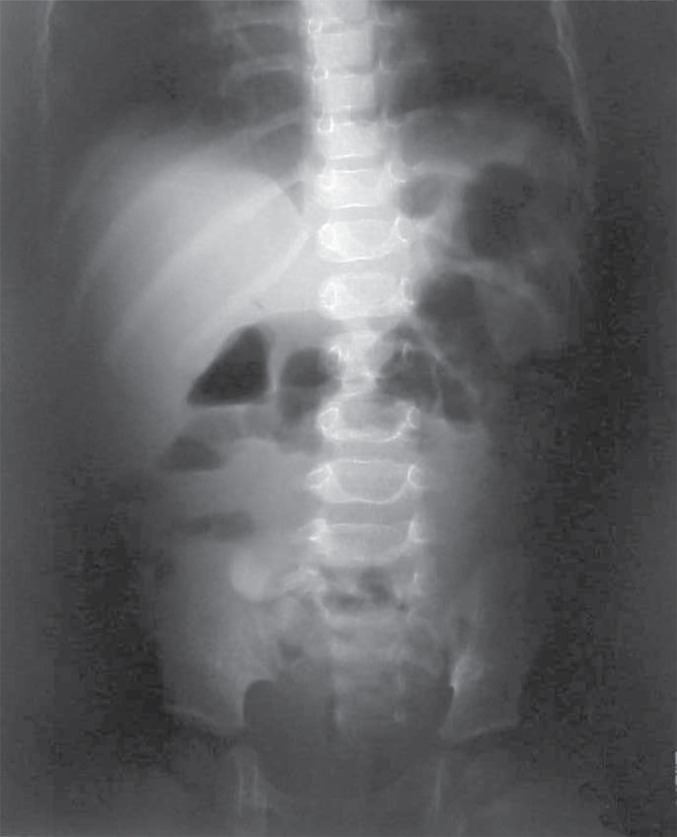
Case No. 1 abdominal X-ray. Imagery was performed two days following hospitalization and revealed a distended left colic flexure.

**Figure 2:**
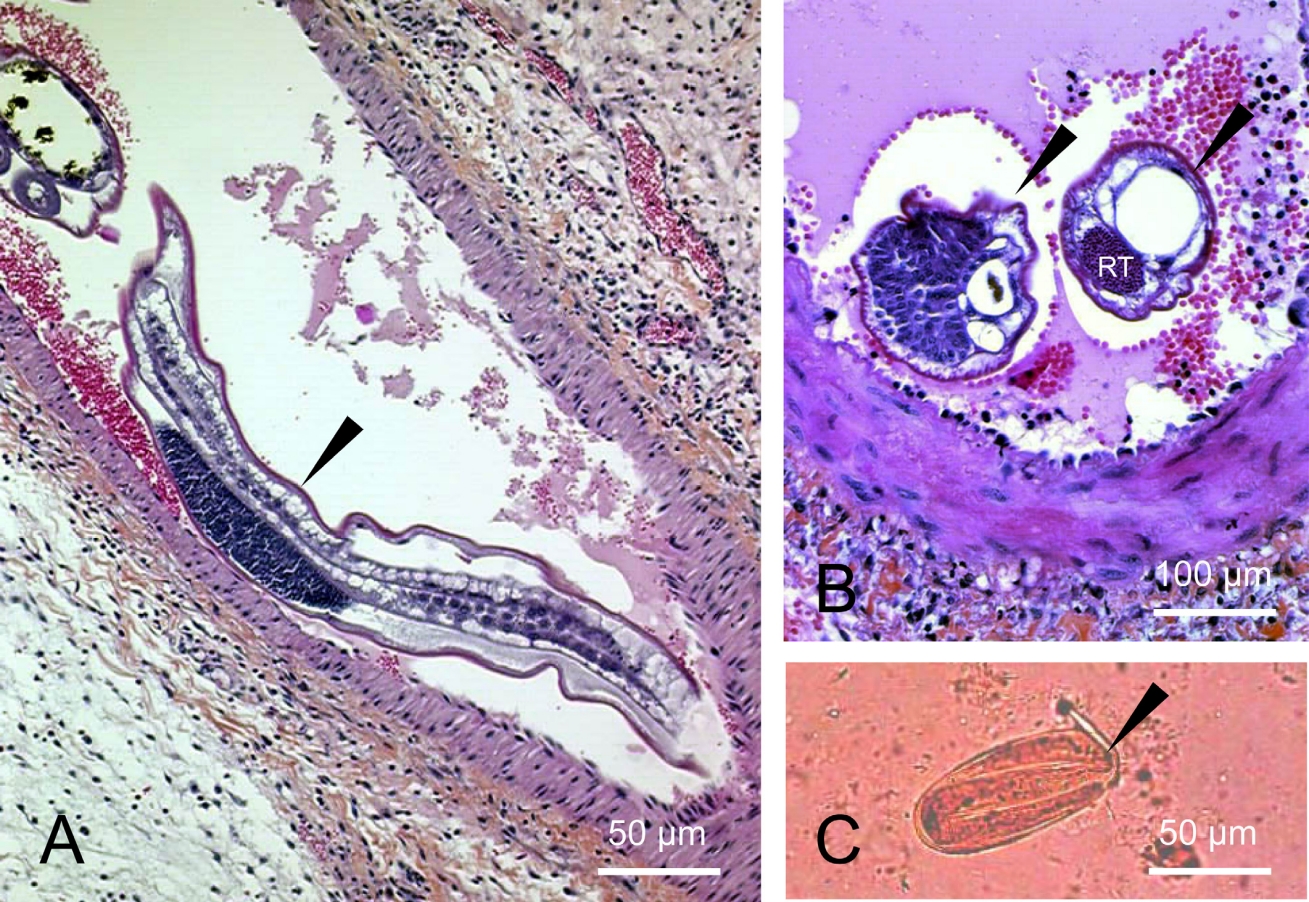
Microscopical aspects of the ileal specimen and parasitic stools examination of case No. 1. A. Longitudinal section of a mesenteric artery with an *A. costaricensis* adult inside arterioles (*dart*) (HES, 100x). B. Cross section of intra-mesenteric arterial adult nematodes (*darts*) with an eosinophilic inflammatory infiltrate in the surrounding tissues. One harbors a reproductive tube (RT) (HES, 100x). C. Impaired embryonated egg of nematode (maybe *A. costaricensis*) (*dart*) measuring 80 × 35 μm (MIF, 200x) found in stools collected four days after abdominal surgery.

**Figure 3:**
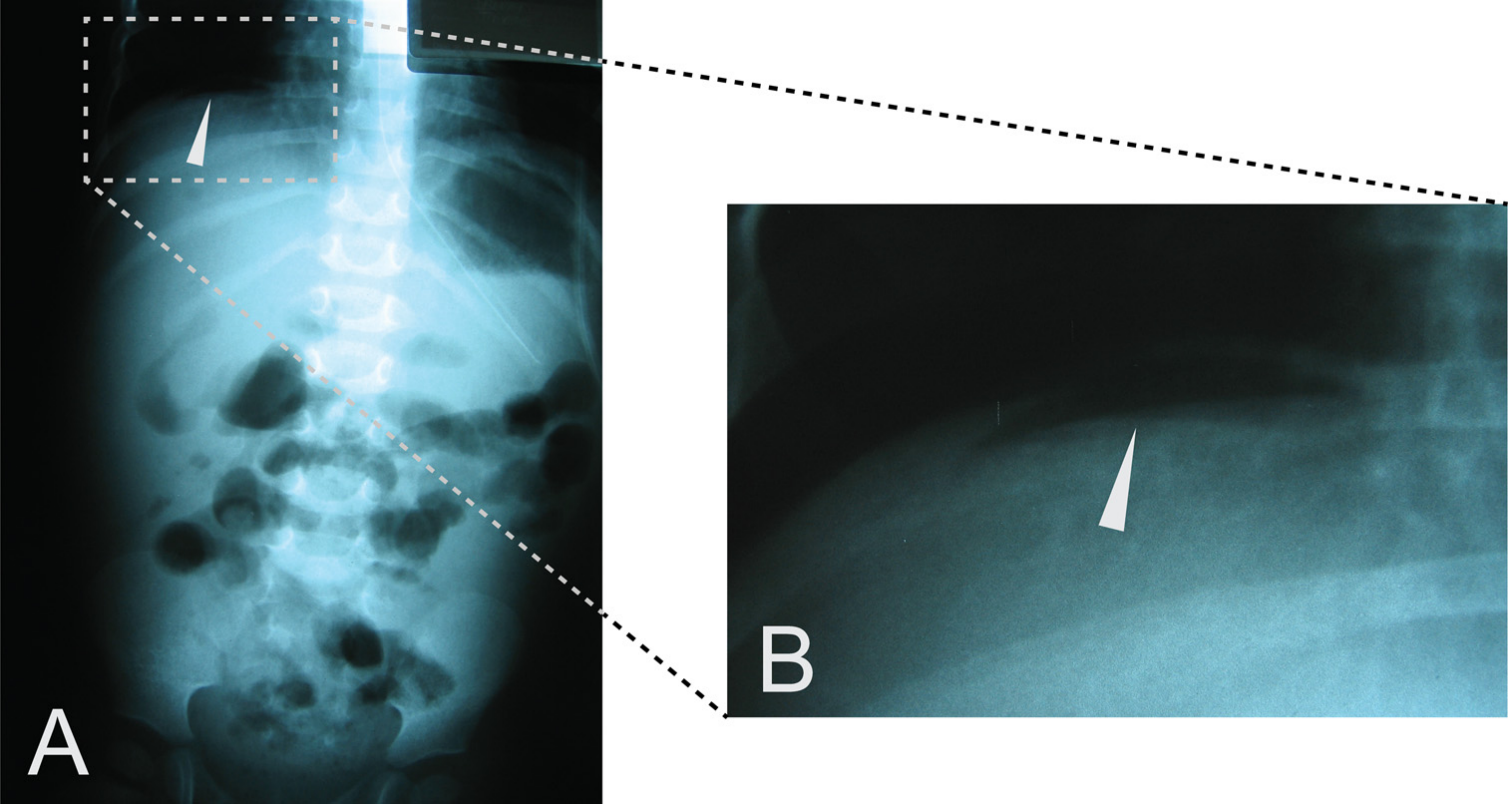
Case No. 2 abdominal X-ray. Imagery was performed 49 days following hospitalization. A. Pneumoperitoneum under the right hypochondria (white arrow). B. Focus on the pneumoperitoneum (white arrow).

**Figure 4:**
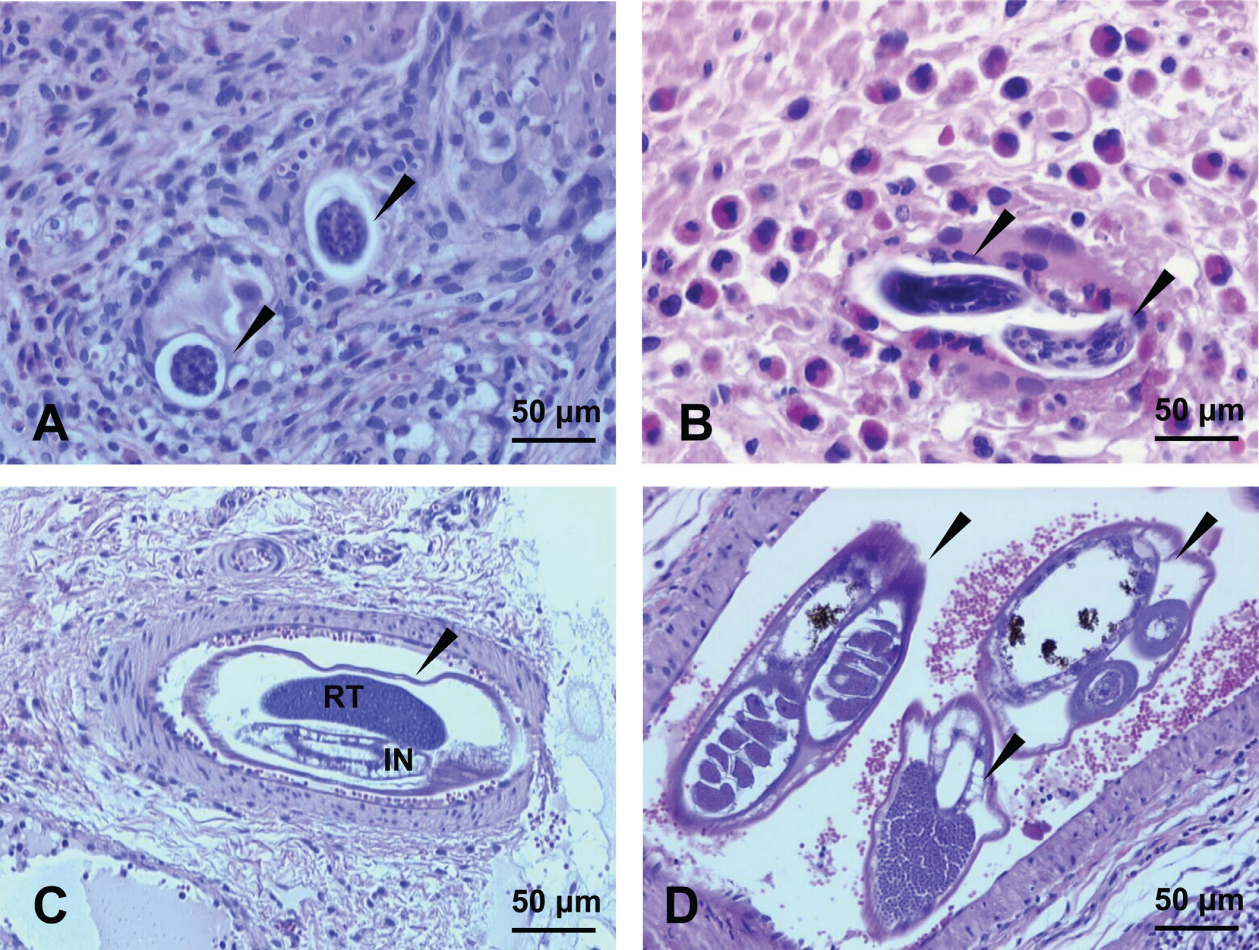
Case No. 2 microscopic aspects of the ileo-colic specimen stained with HES. A. Section of the intestine showing two thin-shelled eggs (*darts*) surrounded by a granulomatous reaction with giant cells (HES, 100x). B. Section of the intestine showing two *A. costaricensis* larvae (*darts*) surrounded by a granulomatous reaction with giant cells and a considerable number of eosinophils cells (HES, 100x). C. Transversal section of a mesenteric artery showing *A. costaricensis* adult worm (*dart*) with a single reproductive tube (RT) and intestine (IN) (HES, 100x). D. Transversal section of a mesenteric small artery showing three sections of adult worms (*darts*) (HES, 100x).

## Discussion

Here, we report two confirmed and two probable cases of HAA in Martinique, thus bringing the total number of HAA cases to 10 in the entire Antilles. Indeed, only six sporadic cases of HAA have been reported in the Antilles over the last two decades. Two cases were diagnosed in travelers returning from the Greater Antilles, one from Puerto Rico [47], and the other from the Dominican Republic [59]. In the Lesser Antilles, one case was reported in Martinique in 1988 in a 16-month-old boy [24], followed by two cases in Guadeloupe in 1987 and 1989 in a 20-month-old and a five year-old, respectively [26], and a presumed case in the Commonwealth of Dominica in a North-American student in 1997 [50]. Clinical and biological features of these cases are summarized in Table 2.

**Table 2:** Abdominal angiostrongyliasis in the Antilles. Literature review of the six HAA cases described in the Greater and Lesser Antilles before the description of the new confirmed and probable HAA cases in Martinique. ND: No Data.

Case	1	2	3	4	5	6
**Background data**						
**Island**	Martinique, Lesser Antilles	Guadeloupe, Lesser Antilles		Dominican Republic, Greater Antilles	Puerto Rico, Greater Antilles	Dominica, Lesser Antilles
**Reference**	[24]	[26]		[59]	[47]	[50]
**Year of diagnosis**	1984	1987	1989	1989	1993	1997
**Season**	Rainy season	ND	ND	ND	ND	ND
**Sex**	M	F	M	M	M	M
**Age**	16 months	20 months	5 years	41 years	42 years	ND (student)
**Area of residence (city)**	Martinique (Sainte-Luce)	ND	ND	ND	Pennsylvania for 2 months, Puerto Rico the past 3 years	US student living in Dominica
**Living conditions**	Rural area, house without water or electricity	Rural & residential area, presence of rats. Wealthy family	Rural area, no water or electricity, presence of rats	ND	ND	ND
**Travel outside of island of residence**	No	ND	ND	ND	Yes, Puerto Rico 2 months before	ND
**Medical history**	None	None	None	ND	ND	ND
**Reported contact with mollusks**	ND	ND	ND	ND	ND	ND
**Clinical picture**						
**Duration of symptoms before admission**	42 days	1 month	3 months	ND	ND	ND
**Extra-digestive symptoms**	Poor general condition, slight fever, constant crying, anorexia	ND	Poor general condition, behavioral disorders, prostration	ND	ND	ND
**Digestive symptoms & bleeding**	Vomiting, melena	Abdominal pain, intestinal occlusion, intermittent rectorrhagia	Intense abdominal pain, diarrhea, melena, rectorrhagia	Recurrent gastro-intestinal bleeding	Severe right-lower quadrant abdominal pain	ND
**Loss of weight**	25%	ND	6 kg	ND	ND	ND
**Laboratory results**						
**Anemia (g/dL)**	6	8.8	4.5	ND	ND	ND
**Mean corpuscular volume (MCV)**	67	ND	88	ND	ND	ND
**Initial WBC (G/L)**	20	19	26.3	ND	ND	ND
**Initial eosinophilia (G/L (%))**	1.46 (7%)	0.38 (2%)	2.49 (9%)	ND	ND	ND
**Max. eosinophilia (G/L)**	2.50	ND	8.41	ND	ND	ND
***Angiostrongylus*** **serodiagnosis**	ND	ND	ND	ND	No	ND
**Abdominal imaging & surgery**						
**Exploratory laparotomy**	Yes	Yes	No	Yes	Yes	ND
**Intestinal resection**	5 cm	18 cm (ileum)	Appendix	ND	12 cm (ileo-ceacum + appendix)	ND
**Histology of resected specimen**						
**Histological examination**	Adults, eggs, and larvae in ileal biopsy	ND	ND	ND	Granulomas, giant cells, eggs, L1 larvae, eosinophilic infiltration	ND
**Diagnosis and medical care**						
**Anthelmintic (Thiabendazole)**	75 mg/kg for 3 days	75 mg/kg for 2 days	75 mg/kg for 3 days, 3 times	ND	ND	ND
**Outcome**	Recovery	Recovery	Recovery	Recovery	Recovery	Recovery

In our case series, the diagnosis of angiostrongyliasis was considered after admission to hospital because of the nonspecific clinical presentation of the disease [33]. Symptoms usually include abdominal pain in the right iliac fossa along with fever, anorexia, vomiting, and persistent eosinophilia (> 2 G/L). The disease is generally mild and self-limiting, but some cases can be complicated by intestinal infarction, pseudo-tumor, acute appendicitis, or digestive perforation, requiring emergency laparotomy and surgical care with an unpredictable prognosis [33].

Typically, diagnosis occurs unexpectedly when an exploratory laparotomy or laparoscopy is required with histological examination of unhealthy tissues. Definitive diagnosis is established when histological examination of resected specimens shows eggs, larvae, or adult parasitic forms in mesenteric arteries [19]. In the absence of parasites, histopathological findings can help the diagnosis when they show granulomatous reactions with massive eosinophilic and giant cell infiltration in the intestinal wall and regional lymph nodes and/or eosinophilic vasculitis of arteries, veins, and lymph vessels [19]. In subclinical forms not requiring laparotomy or surgery, diagnosis may be established when IgG anti-crude adult worm antigens are found by ELISA-based serological analysis, but such analyses are available in only a few laboratories worldwide [1,3,15,48,67]. Serodiagnosis of *A. costaricensis* is somewhat unsatisfactory because of cross-reactions with *A. cantonensis, Strongyloides stercoralis*, and *Gnathostoma spinigerum* [47]. Better specificity is observed when antigens are derived from *A. costaricensis* eggs or the reproductive organs of females [4,15]. New tools are now being used to improve the diagnosis in countries in which the disease is endemic, such as Brazil, particularly PCR on paraffin-embedded biopsy tissue or sera, which can lead to a 20% increase in the rate of presumptive diagnoses [8,53]. Unfortunately, such biological tools are not yet available in the French territories.

In our study, the pediatric cases (cases 1 and 2) illustrate the severe and chronic form of the disease, with necrotizing intestinal inflammation, requiring laparotomy and partial intestinal resection. These two cases were characterized by weight loss, anemia, and a long hospital stay (Table 1), consistent with the results observed in the three reported pediatric cases in Martinique and Guadeloupe in the 1980's [24,26]. In both of our pediatric cases, examination of the ileo-cecal surgical specimen unexpectedly led to the diagnosis of HAA through microscopic identification of *A. costaricensis* eggs and larvae in the context of a typical, intense ileo-cecal inflammatory, eosinophilic, and granulomatous reaction. Case 1 was particularly intriguing due to the presence of degenerated helminth eggs in the feces after surgery (Fig. 2). We could not confirm them as *A. costaricensis* eggs based solely on morphological observation and molecular investigation was not performed. Indeed, detection of *A. costaricensis* eggs in feces has rarely been described, since their elimination is prevented by the inflammatory reaction in the intestinal wall. However, in this case, surgery may have liberated the eggs in the digestive tract. Cases 3 and 4 illustrate the presumptive and probable diagnoses of less severe forms of HAA, based solely on abdominal symptoms and marked eosinophilia. The absence of histopathological examination of digestive specimens and specific *A. costaricensis* serological and PCR tests in the French territory hampered a definitive diagnosis. The main elements supporting the diagnosis of HAA were the positive results to *A. cantonensis* serological tests (which are often cross-reactive *A. costaricensis* antigens), combined with negative results for serological tests for other parasites. Several negative parasitological examinations of feces and the absence of headaches and neurological symptoms, respectively ruled out a possible differential diagnosis of strongyloidiasis and angiostrongyliasis due to *A. cantonensis.* All patients recovered without sequelae.

There is no consensus concerning the treatment of HAA [38]. It is mainly supportive, focusing on analgesia, hydration, and nutrition. Surgery can solve ischemia-related intestinal damage and perforation. Anthelmintic treatment using benzimidazole-derived compounds is debatable because their larvicidal effect aggravates the inflammatory response, leading to more severe lesions, and may favor the erratic migration of adult parasites and larvae [39]. Recent studies in mouse models showed that prophylactic enoxaparin treatment does not prevent tissue damage and mortality related to abdominal angiostrongyliasis [54,55]. The four patients in our case series were treated with an anthelmintic as standard treatment for cases of high eosinophilia before diagnostic confirmation.

The mode of transmission of HAA varies depending on the geographical area, generally through the slime of mollusks (*i.e*., mollusks mouthed by young children or in poorly washed vegetables or aromatic plants) or the consumption of raw mollusks (*i.e.*, during atypical medicinal practices) [28]. The mode of transmission for the two confirmed cases is unclear, as no evident contact with mollusks was reported for either patient. However, environmental investigation in one case found the frequent presence of slugs (und. species) near the house, sometimes reaching the bathroom, and the frequent presence of *Achatina fulica* snails in their favorite strolling zone in the Morne-Rouge district. In the other case, the parents did not exclude contact between their child and mollusks, but no specific event was reported. The modest family house was in a district infested with slugs and snails during the rainy season, including *Limicolaria aurora* and *A. fulica*, and surrounded by brush and sugar cane fields with many rodents. These mollusks were not examined to ascertain the presence of *A. costaricensis*. Aside from the adult case diagnosed during the dry season in February 2017, all diagnoses in children were made during the rainy season, when slugs and snails are abundant. Environmental studies are needed to better understand the routes of HAA transmission and evaluate the infection rate and dissemination in mollusks and rodents in Martinique.

The only environmental investigation in the French Antilles was conducted on *Rattus rattus* and *Rattus norvegicus* in Guadeloupe (an island close to Martinique) in 1992 and showed that 7.5% of rats tested were naturally infected by *A. costaricensis* [25]. These rat species are also found in Martinique and may be the main definitive hosts there [49] (Table 3). Among the most common definitive hosts in South America, the rodent families Cricetidae and Heteromyidae are absent in Martinique [52,61,63]. Slugs, acting as intermediate hosts for *A. costaricensis* in South America, are also found in Martinique, including the Veronicellidae family (*Sarasinula plebeia*, *Diplosolenodes occidentalis*) and limacid slugs [10,62,63] (Table 3). The aquatic snails *Biomphalaria glabrata* and *B. straminea* could have been a potential intermediate host, but are now considered to have been eradicated in Martinique, following a control program on intestinal parasitosis initiated in 1978 [10,22] (Table 3). Finally, *A. fulica* snails are not considered to be major intermediate hosts in the wild, although they are capable of hosting *A. costaricensis* larvae in laboratory models [6]. This invasive species, first described in 1989 in Martinique, is responsible for the emergence of central nervous system angiostrongyliasis due to *Angiostrongylus cantonensis* in the Lesser Antilles [9].

**Table 3:** *A. costaricensis* definitive and intermediate hosts described in the literature and comparison with the species found in Martinique. The definitive hosts of *A. costaricensis* in Martinique could be the rodent species *Rattus rattus* and *Rattus norvegicus*. The intermediate hosts could be *Sarasinula plebeia*, *Diplosolenodes occidentalis*, *Deroceras laeve*, and *Biomphalaria* spp.

*Definitive hosts*				

Order	Family	Species	Found in Martinique	Countries & references
Rodentia	Cricetidae	*Sigmodon hispidus*	No	Costa Rica [42], Panama [63], United States [65]
		*Oligoryzomys (=Oryzomys) fulvescens*	No	Panama [63]
		*Sooretamys angouya (=Oryzomys ratticeps)*	No	Brazil [20]
		*Oligoryzomys nigripes (=Oryzomys eliurus)*	No	Brazil [20]
		*Zygodontomys microtinus*	No	Panama [63]
		*Oryzomys caliginosus*	No	Colombia [35]
		*Akodon montensis*	No	Argentina [52]
	Muridae	*Rattus rattus*	Yes	Costa Rica [42], Panama [63], Guadeloupe [25]
		*Rattus norvegicus*	Yes	Guadeloupe [25]
	Heteromyidae	*Liomys adspersus*	No	Panama [63]
	Echimyidae	*Proechimys* sp.	No	Venezuela [56]
Carnivora	Procyonidae	*Nasua narica bullata*	No	Costa Rica [41,42,57]
		*Procyon lotor*	No	United States [40]
Didelphimorphia	Didelphidae	*Didelphis virginiana *	No	United States [40]
Primates	Hylobatidae	*Hylobates syndactylus*	No	United States [40]
	Aotidae	*Aotus nancymaae*	No	United States [40]
	Cebidae	*Saguinus mystax*	No	Peru [60]

*Intermediate hosts (Gastropods)*				

Class	Family	Species	Found in Martinique	Countries & references
Gastropods: slugs	Veronicellidae	*Sarasinula pleibeia* *(= Vaginulus plebeius)*	Yes	Brazil [29], Nicaragua [12]
		*Diplosolenodes occidentalis*	Yes	Honduras [5]
		*Belocaulus angustipes*	No	Brazil [51]
		*Phyllocaulis variegatus*	No	Brazil [21,51]
		*Phyllocaulis soleiformis*	No	Brazil [51]
		*Sarasinula marginata*	No	Brazil [31,37]
	Limacidae	*Limax maximus*	No	Brazil [62]
		*Limax flavus*	No	Brazil [62]
	Agriolimacidae	*Deroceras laeve*	Yes	Brazil [36]
Gastropods: snails	Bradybaenidae	*Bradybaena similaris*	No	Brazil [51]
	Strophocheilidae	*Megalobulimus abbreviatus *	No	Brazil [62]
	Planorbidae	*Biomphalaria glabrata*	Considered eradicated from Martinique	Brazil [22,32]
		*Biomphalaria straminea*	Yes	Brazil [32]
		*Biomphalaria tenagophila*	No	Brazil [2,32]
	Achatinidae	*Achatina fulica*^a^	Yes	Brazil [6,16]

a *Achatina fulica* snails are not considered to be major intermediate hosts in the wild.

HAA is an emerging parasitic disease in the neotropics, which is not critical in most cases, but nonetheless potentially life-threatening. In Martinique, this zoonosis is sporadic and rare, with an estimated incidence of 0.003 cases/100,000 habitants/year in this study. However, HAA can be misdiagnosed due to its nonspecific clinical presentation, paucisymptomatic cases, and the lack of awareness and information in the medical community concerning this disease [18]. Eosinophilia of undetermined origin is often treated using empirical anthelmintic treatment (generally a combination of albendazole or flubendazole and ivermectin) to cover a broad range of parasitic disease etiologies known in Martinique, including ascariasis, enterobiasis, strongyloidiasis, trichuriasis, and ankylostomiasis [14]. The combination of abdominal pain and hypereosinophilia should suggest potential HAA disease as for other well-known intestinal helminthiases, and clinicians should then seek histological or biological evidence of HAA. Thus, efforts should aim to raise awareness in the medical community and facilitate access to diagnostic tools, including serodiagnosis and PCR-based methods. An epidemiological study focusing on intermediate hosts would lead to a better understanding of disease transmission in Martinique and help establish more efficient prophylactic measures.

## References

[1] Abrahams-Sandi E, Mesén-Ramírez P, Suarez-Chacón D, Fernández-Quesada K. 2011 An indirect immunofluorescence antibody test employing whole eggs as the antigen for the diagnosis of abdominal angiostrongyliasis. Memórias do Instituto Oswaldo Cruz, 106, 390–393. 21739024 10.1590/s0074-02762011000400002

[2] Banevicius NMS, Zanotti-Magalhães EM, Magalhães LA, Linhares AX. 2006 Behavior of *Angiostrongylus costaricensis* in planorbids. Brazilian Journal of Biology, 66, 199–204. 10.1590/s1519-69842006000200003 16710513

[3] Ben R, Rodrigues R, Agostini AA, Graeff-Teixeira C. 2010 Use of heterologous antigens for the immunodiagnosis of abdominal angiostrongyliasis by an enzyme-linked immunosorbent assay. Memórias do Instituto Oswaldo Cruz, 105, 914–917. 21120363 10.1590/s0074-02762010000700013

[4] Bender AL, Maurer RL, da Silva MCF, Ben R, Terraciano PB, da Silva ACA. 2003 Eggs and reproductive organs of female *Angiostrongylus costaricensis* are more intensely recognized by human sera from acute phase in abdominal angiostrongyliasis. Revista da Sociedade Brasileira de Medicina Tropical, 36, 449–454. 12937720 10.1590/s0037-86822003000400003

[5] Caballero R, Thomé JW, Andrews KL, Rueda A. 1991 Babosas de Honduras (Soleolifera: Veronicellidae): biología, ecología, distribución, descripción, importancia económica, y claves para su identificación. Ceiba, 32, 107–125.

[6] Carvalho O dos S, Teles HM, Mota EM, Lafetá C, de Mendonça GF, Lenzi HL. 2003 Potentiality of *Achatina fulica* Bowdich, 1822 (Mollusca: Gastropoda) as intermediate host of the *Angiostrongylus costaricensis* Morera & Céspedes 1971. Revista da Sociedade Brasileira de Medicina Tropical, 36, 743–745. 15049117 10.1590/s0037-86822003000600017

[7] Cespede R, Salas J, Mekbel S, Troper L, Mullner F, Morera P. 1967 Granulomas entéricos y linfaticos con intensa eosinofilia tisular producidos por um estrongilideo (Strongylata). Acta Médica Costarricense, 10, 235–255.

[8] Da Silva ACA, Graeff-Teixeira C, Zaha A. 2003 Diagnosis of abdominal angiostrongyliasis by PCR from sera of patients. Revista do Instituto de Medicina Tropical de São Paulo, 45, 295–297. 14743672 10.1590/s0036-46652003000500011

[9] Dard C, Piloquet J-E., Qvarnstrom Y, Fox LM, M'kada H, Hebert J-C., Mattera D, Harrois D. 2017 First evidence of angiostrongyliasis caused by *Angiostrongylus cantonensis* in Guadeloupe, Lesser Antilles. American Journal of Tropical Medicine and Hygiene, 96, 692–697. 28070007 10.4269/ajtmh.16-0792 PMC5361547

[10] Dellanoye R, Charles L, Pointier J, Massemin D. 2015. *Sarasinula plebeia*, in Mollusques continentaux de la Martinique, Collection Inventaires et biodiversité. Biotope Éditions & Muséum National d'Histoire Naturelle: Mèze & Paris. p. 328.

[11] Duarte Z, Morera P, Davila P, Gantier JC. 1992 *Angiostrongylus costaricensis* natural infection in *Vaginulus plebeius* in Nicaragua. Annales de Parasitologie Humaine et Comparée, 67, 94–96. 1290381 10.1051/parasite/199267394

[12] Duarte Z, Morera P, Vuong PN. 1991 Abdominal angiostrongyliasis in Nicaragua: a clinico-pathological study on a series of 12 cases reports. Annales de Parasitologie Humaine et Comparée, 66, 259–262. 1822656 10.1051/parasite/1991666259

[13] Eamsobhana P, Gan XX, Ma A, Wang Y, Wanachiwanawin D, Yong HS. 2014 Dot immunogold filtration assay (DIGFA) for the rapid detection of specific antibodies against the rat lungworm *Angiostrongylus cantonensis* (Nematoda: Metastrongyloidea) using purified 31-kDa antigen. Journal of Helminthology, 88, 396–401. 23710755 10.1017/S0022149X13000321

[14] Edouard A, Edouard S, Desbois N, Plumelle Y, Rat C, Calès-Quist D. 2004 Évolution de la prévalence des parasitoses digestives au CHU de Fort-de-France (Martinique). Presse Médicale, 33, 707–709. 10.1016/s0755-4982(04)98725-8 15257226

[15] Geiger SM, Laitano AC, Sievers-Tostes C, Agostini AA, Schulz-Key H, Graeff-Teixeira C. 2001 Detection of the acute phase of abdominal angiostrongyliasis with a parasite-specific IgG enzyme linked immunosorbent assay. Memórias do Instituto Oswaldo Cruz, 96, 515–518. 11391424 10.1590/s0074-02762001000400012

[16] Graeff-Teixeira C. 2007 Expansion of *Achatina fulica* in Brazil and potential increased risk for angiostrongyliasis. Transactions of the Royal Society of Tropical Medicine and Hygiene, 101, 743–744. 17481682 10.1016/j.trstmh.2007.03.012

[17] Graeff-Teixeira C, Agostini AA, Camillo-Coura L, Ferreira-da-Cruz MF. 1997 Seroepidemiology of abdominal angiostrongyliasis: the standardization of an immunoenzymatic assay and prevalence of antibodies in two localities in southern Brazil. Tropical Medicine & International Health, 2, 254–260. 9491104 10.1046/j.1365-3156.1997.d01-266.x

[18] Graeff-Teixeira C, Camillo-Coura L, Lenzi HL. 1987 Abdominal angiostrongyliasis − an under-diagnosed disease. Memórias do Instituto Oswaldo Cruz, 82, 353–354. 3509192 10.1590/s0074-02761987000800068

[19] Graeff-Teixeira C, Camillo-Coura L, Lenzi HL. 1991 Histopathological criteria for the diagnosis of abdominal angiostrongyliasis. Parasitology Research, 77, 606–611. 1792232 10.1007/BF00931023

[20] Graeff-Teixeira C, de Avila-Pires FD, Machado R de C, Camillo-Coura L, Lenzi HL. 1990 Identification of wild rodents as hosts of *Angiostrongylus costaricencis* in southern Brazil. Revista do Instituto de Medicina Tropical de São Paulo, 32, 147–150. 2135366

[21] Graeff-Teixeira C, Thomé JW, Pinto SC, Camillo-Coura L, Lenzi HL. 1989 *Phyllocaulis variegatus*-an intermediate host of *Angiostrongylus costaricensis* in south Brazil. Memórias do Instituto Oswaldo Cruz, 84, 65–68. 2319952 10.1590/s0074-02761989000100012

[22] Guerino LR, Carvalho JF, Magalhães LA, Zanotti-Magalhães EM. 2017 Susceptibility of *Biomphalaria glabrata* submitted to concomitant infection with *Angiostrongylus costaricensis* and *Schistosoma mansoni*. Brazilian Journal of Biology, 77, 451–458. 10.1590/1519-6984.15215 27683809

[23] Incani RN, Caleiras E, Martín M, González C. 2007 Human infection by *Angiostrongylus costaricensis* in Venezuela: first report of a confirmed case. Revista do Instituto de Medicina Tropical de São Paulo, 49, 197–200. 17625700 10.1590/s0036-46652007000300012

[24] Jeandel R, Fortier G, Pitre-Delaunay C, Jouannelle A. 1988 Angiostrongylase intestinale à *Angiostrongylus costaricencis*. À propos d'un cas en Martinique. Gastroentérologie Clinique et Biologique, 12, 390–393. 3384256

[25] Juminer B, Borel G, Mauleon H, Durette-Desset MC, Raccurt CP, Roudier M. 1993 L'infection murine naturelle par *Angiostrongylus costaricensis* Morera et Céspedes, 1971 à la Guadeloupe. Bulletin de la Société de Pathologie Exotique, 86, 502–505. 7819811

[26] Juminer B, Roudier M, Raccurt CP, Pujol HP, Gerry F, Bonnet R. 1992 Présence d'angiostrongylose en Guadeloupe. À propos de deux cas récents. Bulletin de la Société de Pathologie Exotique, 85, 39–43. 1596956

[27] Kaminsky R, Caballero R, Andrews K. 1995 Presencia de *Angiostrongylus costaricensis* en Honduras y sus relaciones agro-ecologicas y humanas. Parasitologia al Dia, 19, 81–90.

[28] Kramer MH, Greer GJ, Quiñonez JF, Padilla NR, Hernández B, Arana BA. 1998 First reported outbreak of abdominal angiostrongyliasis. Clinical Infectious Diseases, 26, 365–372. 9580096 10.1086/516325

[29] Laitano AC, Genro JP, Fontoura R, Branco SS, Maurer RL, Graeff-Teixeira C. 2001 Report on the occurrence of *Angiostrongylus costaricensis* in southern Brazil, in a new intermediate host from the genus *Sarasinula* (Veronicellidae, Gastropoda). Revista da Sociedade Brasileira de Medicina Tropical, 34, 95–97. 11340504 10.1590/s0037-86822001000100015

[30] Lasso R. 1985. Angiostrongiliasis en Ecuador. Universidad de Guayaquil Comisión de Ciencia y Tecnología Boletín informativo. N. 3.

[31] Lima LC, Massara CL, de Souza CP, Jannotti-Passos LK, Lenzi HL. 1992 *Sarasinula marginata* (Semper, 1885) (Mollusca, Soleolifera) from Belo Horizonte (MG, Brasil) as a potential intermediate host of *Angiostrongylus costaricensis* Morera, Cespedes, 1971. Revista do Instituto de Medicina Tropical de São Paulo, 34, 117–122 1340024 10.1590/s0036-46651992000200006

[32] Lima LC, Massara CL, de Souza CP, Vidigal TD, Lenzi HL, Carvalho O dos S. 1992 The susceptibility of Planorbidae from the metropolitan area of Belo Horizonte, MG (Brazil) to *Angiostrongylus costaricensis* (Nematoda, Angiostrongylidae). Revista do Instituto de Medicina Tropical de São Paulo, 34, 399–402. 1342102 10.1590/s0036-46651992000500005

[33] Loría-Cortés R, Lobo-Sanahuja JF. 1980 Clinical abdominal angiostrongylosis. A study of 116 children with intestinal eosinophilic granuloma caused by *Angiostrongylus costaricensis*. American Journal of Tropical Medicine and Hygiene, 29, 38–44. 7406104

[34] Maldonado A, Simoes R, Thiengo S. 2012. Angiostrongyliasis in the Americas, in Zoonosis, Editor. Lorenzo-Morales J, Rijeka, Croatia. p. 303-320.

[35] Malek EA. 1981 Presence of *Angiostrongylus costaricensis* Morera and Céspedes 1971 in Colombia. American Journal of Tropical Medicine and Hygiene, 30, 81–83. 7212175 10.4269/ajtmh.1981.30.81

[36] Maurer RL, Graeff-Teixeira C, Thome JW, Chiaradia LA, Sugaya H, Yoshimura K. 2002 Natural infection of *Deroceras laeve* (Mollusca: gastropoda) with metastrongylid larvae in a transmission focus of abdominal angiostrongyliasis. Revista do Instituto de Medicina Tropical de São Paulo, 44, 53–54 11896413 10.1590/s0036-46652002000100009

[37] Mendonca CLGF, Carvalho OS, Lenzi HL. 2002 *Angiostrongylus costaricensis* life cycle in the intermediate host *Sarasinula marginata* Semper, 1885 (Mollusca: Soleolifera). Revista da Sociedade Brasileira de Medicina Tropical, 35, 199–200. 12011932 10.1590/s0037-86822002000200013

[38] Mentz MB, Graeff-Teixeira C. 2003 Drug trials for treatment of human angiostrongyliasis. Revista do Instituto de Medicina Tropical de São Paulo, 45, 179–184. 14502343 10.1590/s0036-46652003000400001

[39] Mentz MB, Graeff-Teixeira C, Garrido CT. 2004 Treatment with mebendazole is not associated with distal migration of adult *Angiostrongylus costaricensis* in the murine experimental infection. Revista do Instituto de Medicina Tropical de São Paulo, 46, 73–75. 15141273 10.1590/s0036-46652004000200003

[40] Miller CL, Kinsella JM, Garner MM, Evans S, Gullett PA, Schmidt RE. 2006 Endemic infections of *Parastrongylus* (=*Angiostrongylus*) *costaricensis* in two species of nonhuman primates, raccoons, and an opossum from Miami, Florida. Journal of Parasitology, 92, 406–408. 16729706 10.1645/GE-653R.1

[41] Monge E, Arroyo R, Solano E. 1978 A new definitive natural host of *Angiostrongylus costaricensis* (Morera and Céspedes 1971). Journal of Parasitology, 64, 34. 627972

[42] Morera P. 1970 Studies of the definitive host of *Angiostrongylus costaricensis* (Morera and Céspedes, 1971). Boletín Chileno de Parasitología, 25, 133–134. 5518359

[43] Morera P. 1985 Abdominal angiostrongyliasis: a problem of public health. Parasitology Today, 1, 173–175. 15275575 10.1016/0169-4758(85)90177-2

[44] Morera P. 1973 Life history and redescription of *Angiostrongylus costaricensis* Morera and Céspedes, 1971. American Journal of Tropical Medicine and Hygiene, 22, 613–621. 4729741 10.4269/ajtmh.1973.22.613

[45] Morera P. 1967 Granulomas entericos y linfaticos con intensa eosinophilia tisular producidos por um estrongilideo (Strongylata; Raillet y Henry, 1913): II. Aspectos parasitologico. Acta Médica Costarricence, 10, 257–265.

[46] Morera P, Andrews KL, Rueda A. 1988 The intermediate host of *Angiostrongylus costaricensis* in Honduras. Revista de Biología Tropical, 36, 575–576. 3273607

[47] Neafie R, Marty A. 1993 Unusual infections in humans. Clinical Microbiology Reviews, 6, 34–56. 8457979 10.1128/cmr.6.1.34 PMC358265

[48] Palominos PE, Gasnier R, Rodriguez R, Agostini AA, Graeff-Teixeira C. 2008 Individual serological follow-up of patients with suspected or confirmed abdominal angiostrongyliasis. Memórias do Instituto Oswaldo Cruz, 103, 93–97. 18327506 10.1590/s0074-02762008005000002

[49] Pascal M, Lorvelec O, Borel G, Rosine A. 2004 Structures spécifiques des peuplements de rongeurs d'agro-écosystèmes et d'écosystèmes “naturels” de la Guadeloupe et de la Martinique. Revue d'Écologie − La Terre et la Vie, 59, 283–292.

[50] Raccurt CP. 1997 Deux angiostrongyloses murines dans la Caraïbe et leurs conséquences humaines : une menace pour Haïti ? Médecine Tropicale : Revue du Corps de Santé Colonial, 57, 412–413. 9612787

[51] Rambo PR, Agostini AA, Graeff-Teixeira C. 1997 Abdominal angiostrongylosis in southern Brazil-prevalence and parasitic burden in mollusc intermediate hosts from eighteen endemic foci. Memórias do Instituto Oswaldo Cruz, 92, 9–14. 10.1590/s0074-02761997000100002 9302406

[52] Robles M del R, Kinsella JM, Galliari C, Navone GT. 2016. New host, geographic records, and histopathologic studies of *Angiostrongylus* spp (Nematoda: Angiostrongylidae) in rodents from Argentina with updated summary of records from rodent hosts and host specificity assessment. Memórias do Instituto Oswaldo Cruz, 111, 181–191. 10.1590/0074-02760150371 PMC4804501 26982178

[53] Rodriguez R, da Silva ACA, Müller CA, Alves SL, Graeff-Teixeira C, Fornari F. 2014 PCR for the diagnosis of abdominal angiostrongyliasis in formalin-fixed paraffin-embedded human tissue. PLoS ONE, 9, e93658. 24705328 10.1371/journal.pone.0093658 PMC3976301

[54] Rodriguez R, Porto SM, Dos Santos Ferrari R, Marcolan AM, da Silva ACA, Graeff-Teixeira C. 2011 Outcomes in mice with abdominal angiostrongyliasis treated with enoxaparin. Parasitology Research, 109, 787–792. 21400113 10.1007/s00436-011-2324-5

[55] Sandri ASS, Rodriguez R, Costa MM, Porto SM, Schwingel D, Vieira MIB. 2018 High-dose enoxaparin in the treatment of abdominal angiostrongyliasis in Swiss mice. Journal of Helminthology, doi: 10.1017/S0022149X17000852. 28969719

[56] Santos CP. 1985 Redescrição de *Angiostrongylus* (*Parastrongylus*) *costaricensis* isolado de novo hospedeiro silvestre, *Proechimys* sp., na Venezuela (Metastrongyloidea, Angiostrongylidae). Memórias do Instituto Oswaldo Cruz, 80, 81–83

[57] Santoro M, Alfaro-Alarcón A, Veneziano V, Cerrone A, Latrofa MS, Otranto D. 2016 The white-nosed coati (*Nasua narica*) is a naturally susceptible definitive host for the zoonotic nematode *Angiostrongylus costaricensis* in Costa Rica. Veterinary Parasitology, 228, 93–95. 27692339 10.1016/j.vetpar.2016.08.017

[58] Sierra E, Morera P. 1968 Angiostrongilosis abdominal. Primer caso humano encontrado em Honduras (Hospital Evangélico de Siguatepeque). Acta Médica Costarricense, 15, 95–99.

[59] Silvera CT, Ghali VS, Roven S, Heimann J, Gelb A. 1989 Angiostrongyliasis: a rare cause of gastrointestinal hemorrhage. American Journal of Gastroenterology, 84, 329–332. 2784029

[60] Sly DL, Toft JD, Gardiner CH, London WT. 1982 Spontaneous occurrence of *Angiostrongylus costaricensis* in marmosets (*Saguinus mystax*). Laboratory Animal Science, 32, 286–288. 6808242

[61] Spratt DM. 2015 Species of *Angiostrongylus* (Nematoda: Metastrongyloidea) in wildlife: A review. International Journal for Parasitology. Parasites and Wildlife, 4, 178–189. 25853051 10.1016/j.ijppaw.2015.02.006 PMC4381133

[62] Teixeira CG, Thiengo SC, Thome JW, Medeiros AB, Camillo-Coura L, Agostini AA. 1993 On the diversity of mollusc intermediate hosts of *Angiostrongylus costaricensis* Morera & Cespedes, 1971 in southern Brazil. Memórias do Instituto Oswaldo Cruz, 88, 487–489. 8107609 10.1590/s0074-02761993000300020

[63] Tesh RB, Ackerman LJ, Dietz WH, Williams JA. 1973 *Angiostrongylus costaricensis* in Panama. Prevalence and pathologic findings in wild rodents infected with the parasite. American Journal of Tropical Medicine and Hygiene, 22, 348–356. 4706428 10.4269/ajtmh.1973.22.348

[64] Ubelaker JE, Hall NM. 1979 First report of *Angiostrongylus costaricensis* Morera and Céspedes 1971 in the United States. Journal of Parasitology, 65, 307. 448616

[65] Vuong PN, Brama P, Bonète R, Houissa-Vuong S, Catanzano-Laroudie M, Baviera E. 2002 Necrotic eosinophilic angiitis with ileal perforation and peritonitis secondary to abdominal angiostrongyliasis. Presse Médicale, 31, 1700–1703. 12467150

[66] Wang Q-P., Lai D-H., Zhu X-Q., Chen X-G., Lun Z-R. 2008 Review: Human angiostrongyliasis. Lancet Infectious diseases, 8, 621–630. 18922484 10.1016/S1473-3099(08)70229-9

[67] Wilkins PP, Qvarnstrom Y, Whelen AC, Saucier C, da Silva AJ, Eamsobhana P. 2013 The current status of laboratory diagnosis of *Angiostrongylus cantonensis* infections in humans using serologic and molecular methods. Hawaii Journal of Medicine & Public Health, 72, 55–57. 23901386 PMC3689480

[68] Wu SS, French SW, Turner JA. 1997 Eosinophilic ileitis with perforation caused by *Angiostrongylus* (*Parastrongylus*) *costaricensis*. A case study and review. Archives of Pathology & Laboratory Medicine, 121, 989–991. 9302934

[69] Zambrano Z. 1973 Ileocolitis pseudotumoral eosinofílica de origen parasitario. Revista Latinoamericana de Patología, 12, 43–50. 4548208

[70] Zavala Velazquez J, Ramirez Baquedano W, Reyes Perez A, BatesFlores M. 1974 Angiostrongilosis costaricensis. Primeros casos mexicanos. Revista de Investigación Clínica, 26, 389–394. 4456513

[71] Zilioto A, Kunzle J, Rus Fernandes L, Prates-Campos C, Britto-Costa R. 1975 Angiostrongilíase: apresentação de um provável caso. Revista do Instituto de Medicina Tropical de São Paulo, 17, 312–318. 1198007

